# Analysing and Correcting the Differences between Multi-Source and Multi-Scale Spatial Remote Sensing Observations

**DOI:** 10.1371/journal.pone.0111642

**Published:** 2014-11-18

**Authors:** Yingying Dong, Ruisen Luo, Haikuan Feng, Jihua Wang, Jinling Zhao, Yining Zhu, Guijun Yang

**Affiliations:** 1 Beijing Research Center for Information Technology in Agriculture, Beijing Academy of Agriculture and Forestry Sciences, Beijing, China; 2 Beijing Research Center for Agricultural Standards and Testing, Beijing Academy of Agriculture and Forestry Sciences, Beijing, China; 3 Institute of Agricultural Remote Sensing & Information Application, Zhejiang University, Hangzhou, Zhejiang, China; 4 LMAM, School of Mathematical Sciences, Peking University, Beijing, China; University of Chinese Academy of Sciences, China

## Abstract

Differences exist among analysis results of agriculture monitoring and crop production based on remote sensing observations, which are obtained at different spatial scales from multiple remote sensors in same time period, and processed by same algorithms, models or methods. These differences can be mainly quantitatively described from three aspects, i.e. multiple remote sensing observations, crop parameters estimation models, and spatial scale effects of surface parameters. Our research proposed a new method to analyse and correct the differences between multi-source and multi-scale spatial remote sensing surface reflectance datasets, aiming to provide references for further studies in agricultural application with multiple remotely sensed observations from different sources. The new method was constructed on the basis of physical and mathematical properties of multi-source and multi-scale reflectance datasets. Theories of statistics were involved to extract statistical characteristics of multiple surface reflectance datasets, and further quantitatively analyse spatial variations of these characteristics at multiple spatial scales. Then, taking the surface reflectance at small spatial scale as the baseline data, theories of Gaussian distribution were selected for multiple surface reflectance datasets correction based on the above obtained physical characteristics and mathematical distribution properties, and their spatial variations. This proposed method was verified by two sets of multiple satellite images, which were obtained in two experimental fields located in Inner Mongolia and Beijing, China with different degrees of homogeneity of underlying surfaces. Experimental results indicate that differences of surface reflectance datasets at multiple spatial scales could be effectively corrected over non-homogeneous underlying surfaces, which provide database for further multi-source and multi-scale crop growth monitoring and yield prediction, and their corresponding consistency analysis evaluation.

## Introduction

Space remote sensing technologies have been widely applied in the research field of agriculture for crop growth parameters estimation, crop growth condition monitoring, and yield evaluation [1]–[3]. Multi-source and multi-scale spatial remote sensing observations provide wealth information for extracting characteristics of crop growth and development with data analysis and mining algorithms and methods [4]–[6]. Due to spatial heterogeneity in crop canopies and diversity of satellite observation systems, differences inevitably exist among analysing results of crop condition monitoring and yield estimation based on multiple remotely sensed observations, which are obtained at different spatial scales from multiple remote sensors during same time periods, and processed by same algorithms, models or methods. Mainly, such differences can be quantitatively described from the following three aspects, i.e. differences of remote sensing observations at multiple spatial scales, different degrees of non-linearity of models and algorithms for crop growth parameters estimation, and spatial scale effects of surface parameters [3], [7]. In this paper, we only discussed about the first factor, i.e. the focus of our research was analysing and correcting the differences among multi-scale spatial remote sensing surface reflectance datasets.

To meet the needs of quantitatively describing space distribution patterns and characteristics, and analysing and correcting differences of physical and mathematical properties and their spatial variations of remote sensing observations, which are obtained at multiple spatial scales from different remote sources, lots of research works have been done based on selecting or constructing statistical or theoretical models and algorithms for data processing [8]–[18]. The biases of mean value, spatial variance, and correlation length of satellite images, and how they change with spatial scale are examined for snow cover patterns analysis, which is shown that it may be difficult to infer the true snow cover variability from the variograms, particularly when they span many orders of magnitude [8]. Bayesian-regularized artificial neural network with data, combined with Moderate Resolution Imaging Spectroradiometer (MODIS) and Multi-angle Imaging Spectroradiometer (MISR), is used for mapping land cover distributions, with application to estimating patterns of deforestation and recovery in Brazil, which yields a quantitative improvement over spectral linear un-mixing of single-angle, multi-spectral data [9]. Precision agriculture management zones are delineated based on years of yield data, and then its scale effect is evaluated from the aspects of relative variance reduction, test of significant differences of the means of yield zones, spatial fragmentation, and spatial agreement. And then, the results show that the post-classification majority filtering eliminates lots of isolated cells or patches caused by random variation while preserving yield means, high variance reduction, general yield patterns, and high spatial agreement [10]. Variogram modeling is applied to evaluate the differences in spatial variability between 8 *km* Advanced Very High Resolution Radiometer (AVHRR), 1 *km* Systeme Probatoire d’Observation de la Terre-Vegetation (SPOT-VGT), and 1 *km*, 500 *m*, and 250 *m* MODIS Normalized Difference Vegetation Index (NDVI) products over eight Earth Observing System (EOS) validation sites, and to characterize the decay of spatial variability as a function of pixel size for spatially aggregated ETM+NDVI products and a real multi-sensor dataset. Then, a new approach is proposed to select the spatial resolution, at which differences in spatial variability between NDVI products from multiple sensors are minimized, and further to provide practical guidance for the harmonization of long-term multi-sensor datasets [11]. Considered spatial heterogeneity of leaf area index (LAI) and non-linearity of LAI inversion models, a new statistical spatial scaling method is proposed to quantitatively analyse scale effects and reveal scaling rules of LAI with ground hyperspectral observations. Numerical results show the spatial consistency of multi-scale estimated LAI after processing with the new proposed scaling method [12]. Also, there are a lot of researches have been done to quantitatively analyse and correct the differences between multi-source and multi-scale spatial remote sensing observations and products [7], [13]–[18].

These existed theoretical models and algorithms for differences analysing and correcting are mainly constructed on the basis of precise math discursion and logical discursion, which confirm their universality theoretically. But actually physical and mathematical properties of underlying surfaces and spatial continuity of research objects cannot meet such preconditions of theoretical models and algorithms strictly. While, for these statistical models and algorithms constructed based on probability theories, though their portability is decreased, but they have better flexibility, practicability, and pertinence compared to theoretical models and algorithms for data processing. Each kind of scheme has both strengths and weaknesses, how to find or construct a new kind of solution for analysing and correcting differences between remote sensing observations at multiple spatial scales, which can meet practical requirements and universal application needs simultaneously, is important for efficiently utilizing multi-source and multi-scale remotely sensed observations for agriculture monitoring and crop production.

In our research, crop canopy is selected as the experimental object. Though crop canopy spectral reflectance as an intrinsic property of crop canopy varies with crop types, the observed reflectance is affected by lots of internal and external factors, when it is measured by remote sensing technologies. For a specific crop type, the remotely sensed crop canopy spectral reflectances are not only influenced by diversity of morphological structure, biochemical and physiological characteristics of crop canopies, but also influenced by soil properties, field management measures, geographical and meteorological conditions, atmospheric environments, solar azimuth and elevation angles, viewing zenith angle, performance of optical remote sensors, earth observing systems, and observing dates and times. So, in actual, there inevitably exist lots of differences between measured canopy reflectances with multiple remote sensors at multiple spatial scales during same time periods. Amounts of existing studies have been done to describe space distribution patterns and characteristics, and to analyse and correct differences of multi-source and multi-scale spatial remote sensing observations with statistical or theoretical models and algorithms. Considering the advantages and disadvantages of statistical and theoretical data processing schemes, we combined these two kinds of schemes together to construct a new method integrating parametric design into statistical theories for multiple spatial remotely sensed surface reflectance datasets processing. This method mainly included extraction and analysis of physical characteristics and mathematical distribution properties, and their spatial variations of multiple surface reflectance datasets, and further differences correction for these multiple observations. Two sets of multiple satellite images obtained in Inner Mongolia and Beijing, China, which were experimental underlying surfaces with different degrees of homogeneity, were selected for new proposed method validation. The results of this research are important for assessing the effectiveness of the new proposed method for use as a tool to analyse and correct multi-scale spatial reflectance datasets differences over non-homogeneous underlying surfaces, and furthermore, they could be used to provide references for multi-scale crop growth monitoring and yield prediction, and to evaluate spatial consistency of multi-scale analysed results in agricultural applications.

## Materials and Methods

### Study Area and Data

**Figure 1:**
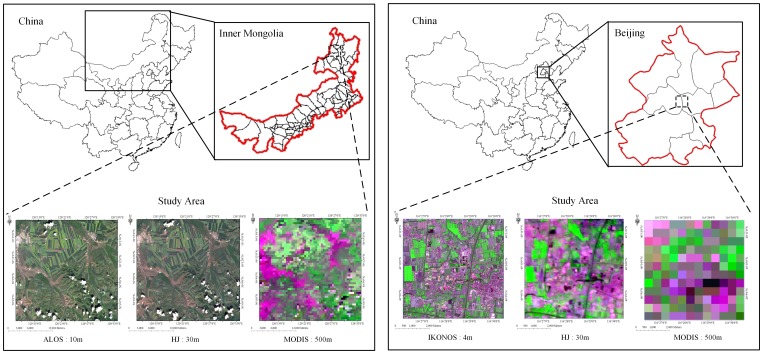
Location of the experimental fields. (A): Location of the experimental field in Labudalin farm of Hailaer Farming Cultivate Bureau in Inner Mongolia, China. (B): Location of the experimental field in Shunyi District and Changping District of Beijing, China.

Two fields with different degrees of homogeneity were selected for numerical experiments in this study. One field was located in Labudalin farm (50°01′ N to 53°26′ N, 119°07′ E to 121°49′ E) of Hailaer Farming Cultivate Bureau in Inner Mongolia, China, which was a farm underlying surface shown in Figure 1A. In this region, there existed lots of concentrated continuous large-scaled farmlands with spatial structure evenly and relatively distributed, so this field could be considered as a homogeneous underlying surface approximately. The main crop in this area was barley, and the main variety managed by normal field management strategies was Kenpimai No. 7, with its seeding time during the period of the end of May and the beginning of June, 2010, and seeding amount around 262.5 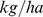. In this experimental field, three multi-source and multi-scale spatial satellite images were obtained during the jointing-booting stage of barley, i.e. Advanced Land Observing Satellite-Advanced Visible and Near Infrared Radiometer type 2 (ALOS-AVNIR2) [19] image with a spatial resolution of 10 *m* obtained in July 8, 2010, Small Remote Sensing Satellite Constellations A Star-CCD2 (HJ 1A-CCD2) [3] image with a spatial resolution of 30 *m* obtained in July 8, 2010, and 8-day composite MODIS Surface Reflectance Product (MOD09A1) [20] with a spatial resolution of 500 *m* obtained during July 4 to 11, 2010. The other field was located in Shunyi District (40°00′ N to 40°18′ N, 116°28′ E to 116°58′ E) and Changping District (40°22′ N to 40°23′ N, 115°50′ E to 116°29′ E) of Beijing, which was a suburban underlying surface shown in Figure 1B. A few of concentrated continuous small-scaled farmlands with unevenly distributed spatial structure existed in this region, so this area could be considered as a non-homogeneous underlying surface. The main crop in this area was winter wheat, and the main varieties managed by normal field management strategies were Lunxuan 987, Zhongmai 12, Zhongmai 11, Jing 9428, Jingdong 8, and Jingdong 12. In this experimental field, three multi-source and multi-scale spatial satellite images were obtained during the flag leaf stage of winter wheat, i.e. IKONOS-Multispectral [3] image with a spatial resolution of 4 *m* obtained in May 6, 2011, Small Remote Sensing Satellite Constellations B Star-CCD2 (HJ 1B-CCD2) [3] image with a spatial resolution of 30 *m* obtained in May 7, 2011, and MOD09A1 with a spatial resolution of 500 *m* obtained during May 1 to 8, 2011.

### Data Pre-Processing

Before analysing and correcting the differences among remote sensing observations at multiple spatial scales, same data pre-processing procedures including radiometric calibration, atmospheric correction, and geometric correction were needed for these spatial observations. Firstly, for radiometric calibration, gains and offsets of remote sensors were used for converting calibrated Digital Numbers (DNs) to absolute units of at-sensor spectral radiance. Secondly, Fast Line-of-sight Atmospheric Analysis of Spectral Hypercubes [21], [22] was selected for atmospheric correction. And then, more than 30 uniformly distributed ground control points in each experimental field were selected for geometric correction. All of the above data pre-processing procedures were conducted in the software Environment for Visualising Images (ENVI, Research Systems Inc. USA).

In this study, croplands in the overlap region of multi-source and multi-scale satellite images were chosen as experimental object. In order to extract croplands, image classification was needed. Taking NDVI as the classification basis, the satellite image with the highest spatial resolution was classified with decision tree classification algorithm in ENVI [23], [24]. Within the time periods of multiple satellite images obtaining, NDVI of barley ranged from 0.40 to 0.95 in Labudalin farm, and NDVI of winter wheat ranged from 0.35 to 0.95 in Shunyi and Changping districts. Then, the classification results were used to mask multiple spatial scale reflectance images for croplands extraction, and then for these non-crop areas, their surface reflectances were set to zero.

### New Data Analysis and Correction Method

In our research, a new method was constructed to analyse and correct the differences among multi-source and multi-scale spatial remotely sensed surface reflectance datasets by integrating parametric design into statistical theories. The main theoretical basis of this proposed method are probability theories and mathematical statistics. According to the fact that multiple spatial canopy reflectance datasets for a specific crop type, such as barley or winter wheat, have different statistical characteristics within the same region, so when we take the crop canopy reflectance as random variables, each of these multi-scale reflectance datasets can be quantitatively described as a normal distribution with its own statistical properties due to the central limit theorem (CLT), which states that, given certain conditions, the arithmetic mean of a sufficiently large number of iterates of independent random variables, each with a well-defined expected value and a well-defined variance, will be approximately normally distributed [25]. Then, multi-source and multi-scale spatial crop canopy reflectance datasets can be approximately taken as multiple normal distributions with different mean values and variances. Furthermore, considering that the crop canopy reflectance are affected by lots of internal and external factors as already mentioned in the introduction, it is necessary to standardize these multiple reflectance distributions first. The data standardization are conducted based on their own mean values and variances, respectively. And, after standardizing these multiple normal distributions, the original multi-source and multi-scale spatial crop canopy reflectance datasets can be approximately described as multiple standard normal distributions with same mean values and variances according to the Lindeberg-Levy central limit theorem [26], which states that, with an increasing volume of canopy spectral data, the standardized sequence of random variable constructed with canopy spectral reflectances will converge to standard normal distribution, which is a Gaussian distribution with 0 as the arithmetic mean, and 1 as the variance. But these multiple standard normal distributions still have differences in distribution shapes, which decided by specific observed multiple spatial reflectance datasets. In probability statistics, if we take the crop canopy reflectance as the sample, then these multi-source and multi-scale reflectance datasets can be taken as sub-samples. According to the law of large numbers (LLN) [27], which describes the result of performing the same experiment a large number of times, the average of the results obtained from a large number of trials should be close to the expected value, and will tend to become closer as more trials are performed. On the basis of LLN, a selected subset of canopy reflectances, with a large enough sample size, obeys the same statistical distribution with the original sample. Theoretically, if the amount of crop canopy spectral reflectances is large enough, multi-source and multi-scale spatial remote sensing observations within the same region should have the same statistical distribution characteristics. With this statistical hypothesis, we can do data correction by taking the spatial observation with highest resolution as the baseline data, i.e. taking the statistical characteristics of Gaussian distribution at the smallest scale as the baseline data to conduct transforming of the other large scale standard normal distributions. Above all, the study of analysing and correcting differences of multiple remotely sensed observations can be converted into data analysis and correction of differences of multiple reflectance distributions.

Before the description of procedures of the new proposed method, we need to firstly introduce five statistical parameters and one function, i.e. arithmetic mean (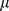), standard deviation (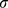), variance (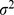), coefficient of skewness (CS), coefficient of kurtosis (CK), and cumulative distribution function (CDF) [3], [18]. Any Gaussian distribution is a version of the standard normal distribution whose domain has been stretched by a factor 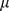 and then translated by a factor 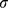, i.e. arithmetic mean and standard deviation can solely determine the Gaussian distribution curve [18]. CDF describes the probability that a real-valued random variable 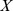 with a given probability distribution will be found at a value less than or equal to 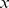. Concept of CDF makes an explicit appearance in statistical analysis in two ways. Cumulative frequency analysis is the analysis of the frequency of occurrence of values of a phenomenon less than a reference value, and empirical distribution function is a formal direct estimate of CDF for which simple statistical properties can be derived and which can form the basis of various statistical hypothesis tests. Such tests can assess whether there is evidence against a sample of data having arisen from a given distribution, or evidence against two samples of data having arisen from the same population distribution [18]. Above all, for the new method, 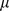 and 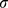 can be used to describe Gaussian distribution characteristics, and CS, CK, and CDF can be used for Gaussian distribution test.

For the new proposed data analysis and correction method, differences of multi-source and multi-scale spatial remote sensing observations were firstly analysed with these above five statistical indexes and CDF, and then method of parametric design was integrated into statistical model for correction of differences between multiple remotely sensed datasets. Taking the spatial observation with highest resolution as the baseline data, the procedures of the new proposed method are listed as below.

- Data initialization.

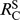
 and 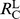 indicate classified crop canopy spectral reflectance datasets at small spatial scale and large spatial scale respectively, where 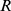 means reflectance, label C means classified data, and “small spatial scale” and “large spatial scale” are abbreviated to S and L respectively. 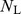 and 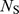 are the numbers of lines and samples of 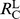 respectively. Let 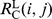 is the 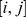 pixel, where 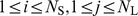, and let 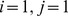 initially. Then, let 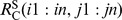 is the corresponding 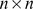 pixel matrix of 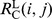 in 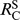. Let 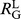 be the data correction result, where label 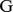 is the abbreviation of Gaussian.

- Data correction.
  - If 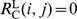, then 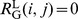 and shift to step C). Otherwise the current process will shift to step b).
  - Firstly, finding the nonzero data in 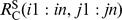 to construct a one-dimensional vector 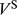, where 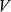 means vector. Secondly, constructing a one-dimensional vector 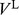, shown in formula (1), where Ones is a matrix with all values as 1, size is a function to extract dimensions of vector. Thirdly, generating a normal distribution one-dimensional vector 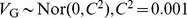, where “
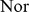
” means normal distribution, and 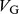 and 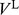 have the same dimensions. Fourthly, calculating the vectors 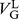, 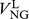, and 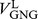, shown in formulas (2), (3), (4) respectively. Where labels NG and GNG mean standard normal distribution and its Gaussian transformation respectively. Among them, 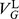 is a variable obeying Gaussian distribution, and 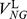 is the standardization of 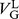, i.e. 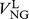 is a standard normally distributed variable, where 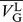 and 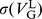 are the arithmetic mean and standard deviation of 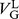, and 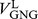 is the transformation of Gaussian distribution, i.e. the standard normal distribution is transformed to a Gaussian distribution taking 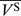 and 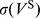 as the arithmetic mean and standard deviation respectively, which are the arithmetic mean and standard deviation of 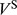 respectively. Finally, set 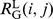 as the value of 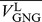, which is the arithmetic mean of 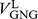.

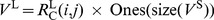
(1)

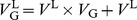
(2)




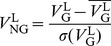
(3)

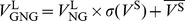
(4)

- Data updating.

If 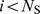, let 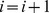 and return the data correction process to step B). Otherwise, if 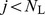, let 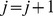, 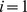, and return the data correction process to step B). Otherwise, if 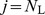 and 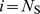, the data correction process ends, and 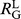 is the correction result.

Above all, the new proposed method has two main components for differences of multi-source and multi-scale spatial remote sensing observations analysing and correcting. On one hand, calculating the statistical parameters and CDF of multi-scale spatial observations allows for the spatial distribution characteristics extraction and Gaussian distribution testing of multi-scale datasets, which provides database for further data correction. On the other hand, data analysis results are taken as the parameters for standardization and transformation of Gaussian distribution, which not only preserves the spatial distribution characteristics at each spatial scale, but also enforces the space consistency of multi-scale observations. So, it can be known that this new method utilising both parametric design method and mathematical model for data analysis and correction is a robust method.

## Results

In this work, two sets of actual multi-source and multi-scale spatial remotely sensed observations in farm underlying surface and suburban underlying surface were chosen for the validation of the new proposed data analysis and correction method. Taken spectral reflectances in red and near infrared as experimental objects, the numerical experiments included multiple optical observations pre-processing, statistical characteristics of multi-scale observations extraction, data correction based on the integration of parameters design method and statistical models, and precision evaluation of the new proposed method in analysing and correcting the differences between multi-scale datasets.

### Farm Underlying Surface

**Figure 2:**
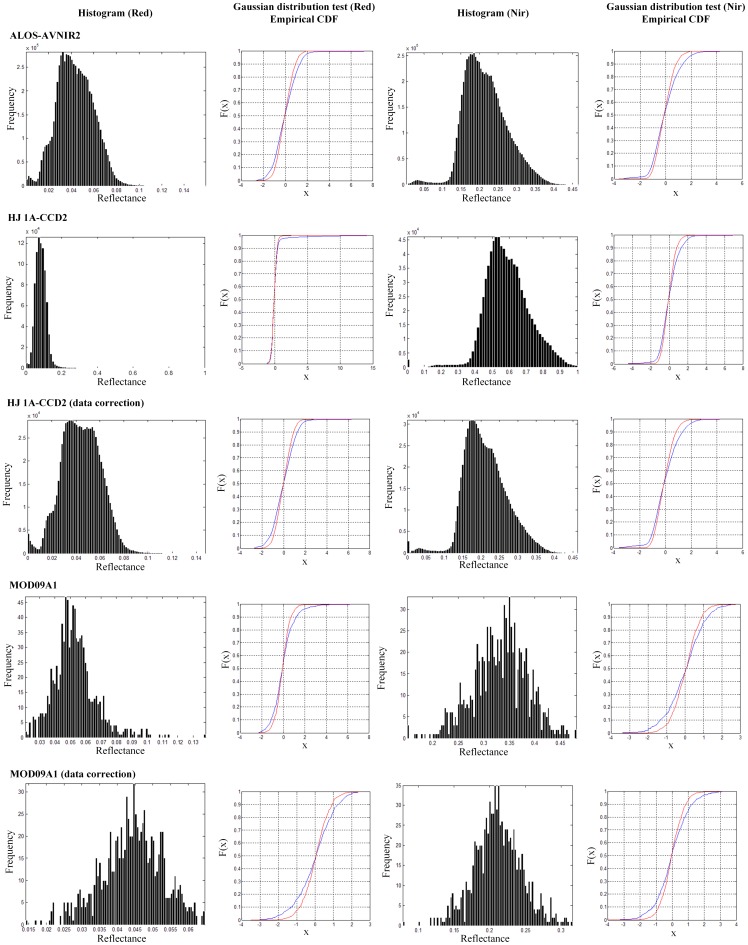
Histograms and Gaussian distribution characteristics of the crop canopy reflectance data at different spatial scales before and after data analysis and correction with the new method in Labudalin farm of Hailaer Farming Cultivate Bureau in Inner Mongolia, China.

In the numerical experiments, multi-source and multi-scale spatial remotely sensed datasets observed in Labudalin farm of Hailaer Farming Cultivate Bureau in Inner Mongolia, China were taken as data source. Firstly, three satellite images, i.e. ALOS-AVNIR2, HJ 1A-CCD2, and MOD09A1, were pre-processed according to the procedures listed in section Data Pre-processing to calculate classified multi-scale crop canopy spectral reflectances. Secondly, five statistical parameters and CDF were calculated to test whether the distributions of red and near infrared spectral reflectances obey Gaussian distributions, and to extract the spatial distribution characteristics of multiple red and near infrared reflectances. Finally, spectral reflectance data of ALOS-AVNIR2 were taken as the baseline data to correct reflectances of HJ 1A-CCD2 and MOD09A1 with the new method. The statistical parameters, histograms and Gaussian distribution characteristics of crop canopy reflectance data at different spatial scales before and after data correction with the new method were shown in Table 1 and Figure 2. In Figure 2, two kinds of CDF curves were involved for Gaussian distribution test. The red CDF curve belonged to the distribution of the sequence of standardized spectral reflectance data, while the blue one was the empirical CDF curve, which belonged to the standard normal distribution.

**Table 1:** Statistical characteristics of the crop canopy reflectance datasets at different spatial scales before and after data analysis and correction with the new method in Labudalin farm of Hailaer Farming Cultivate Bureau in Inner Mongolia, China.

Spectral range	Statistical parameters	ALOS-AVNIR2	HJ 1A-CCD2	HJ 1A-CCD2 (data correction)	MOD09A1	MOD09A1 (data correction)
Red						
	*μ*	0.043538	0.091613	0.044405	0.052868	0.044552
	*σ*	0.015953	0.073000	0.016235	0.013671	0.008568
	*σ* ^2^	0.000255	0.005329	0.000264	0.000187	0.000073
	CS	0.205227	7.668312	0.101705	1.049189	-0.291990
	CK	0.058132	76.130234	0.250088	3.179078	0.019312
Nir						
	*μ*	0.216418	0.592245	0.211834	0.332550	0.210138
	*σ*	0.058143	0.133190	0.059050	0.053253	0.033355
	*σ* ^2^	0.003381	0.017740	0.003487	0.002836	0.001113
	CS	0.260935	0.242875	0.134787	−0.238342	0.039368
	CK	0.614903	2.269275	0.948449	0.203455	0.501980

From Table 1 and Figure 2, we concluded that, except the red reflectance of HJ 1A-CCD2 was found to have a little leptokurtic distribution, the other red and near infrared reflectances of ALOS-AVNIR2, HJ 1A-CCD2, and MOD09A1 all obeyed Gaussian distribution approximatively, though there existed some small differences among the statistical characteristics of these Gaussian distributions. Then, the new method was selected for these differences correction, with ALOS-AVNIR2 data as small scale image, and HJ 1A-CCD2 and MOD09A1 data as large scale images. Based on the analysis of the statistical characteristics of corrected reflectance datasets, we knew that the Gaussian distribution of satellite images at three different spatial scales were almost the same. Taken red reflectance of HJ 1A-CCD2 as an example, after data analysis and correction, its arithmetic mean value changed from 0.091613 to 0.044405, which was more closer to the arithmetic mean value of red reflectance of ALOS-AVNIR2, i.e. 0.043538, and standard deviation value changed from 0.073000 to 0.016235, which was more closer to the standard deviation value of red reflectance of ALOS-AVNIR2, i.e. 0.015953. And the CDF curve of corrected HJ 1A-CCD2 was more similar to the CDF curve of ALOS-AVNIR2. Also similar analysed results were achieved for near infrared reflectance of HJ 1A-CCD2, and red and near infrared reflectances of MOD09A1. That meant these multi-scale spatial reflectances had similar Gaussian distribution properties after data processing with the new method. Above all, for this farm underlying surface, which was an approximately homogeneous underlying surface, the new proposed method not only effectively reduced the differences between multi-source and multi-scale spatial observations, but also had strong adaptability and high correction accuracy.

### Suburban Underlying Surface

**Figure 3:**
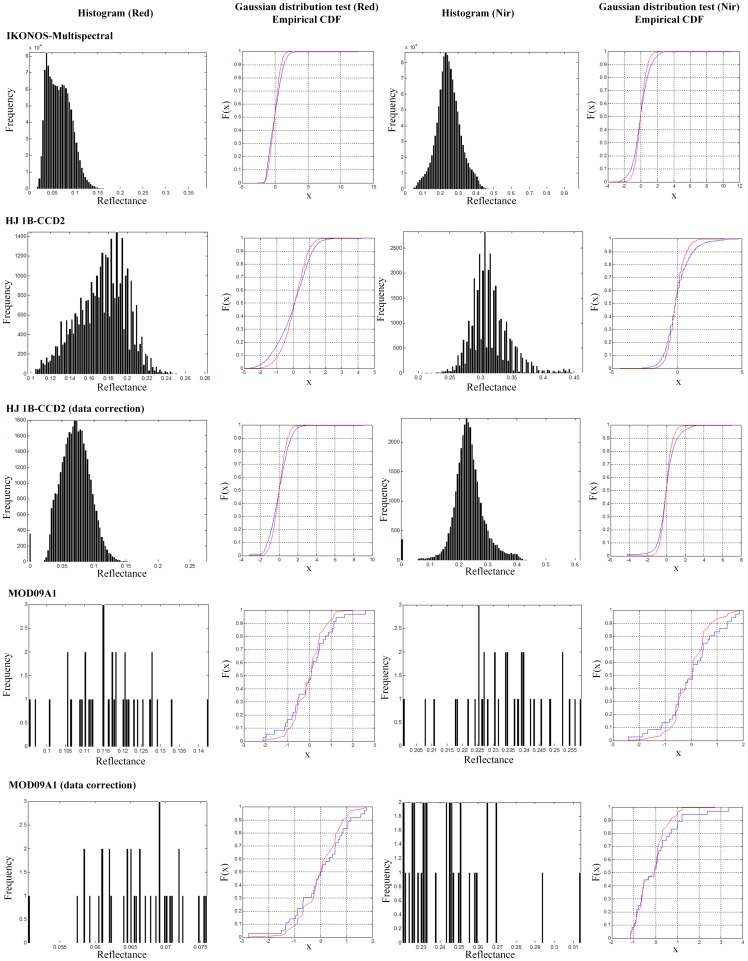
Histograms and Gaussian distribution characteristics of the crop canopy reflectance data at different spatial scales before and after data analysis and correction with the new method in Shunyi District and Changping District of Beijing, China.

Multiple spatial remote sensing images IKONOS-Multispectral, HJ 1B-CCD2, and MOD09A1 observed in Shunyi District and Changping District of Beijing, China were taken as data source for numerical experiments. Firstly, three satellite images were pre-processed to calculate classified reflectances. Secondly, statistical parameters and CDF were calculated to test whether the distribution of reflectances obey Gaussian distributions, and to extract distribution characteristics of multi-scale spatial red and near infrared spectral reflectances respectively. Finally, IKONOS-Multispectral were taken as the baseline data to correct reflectances of HJ 1B-CCD2 and MOD09A1. The statistical parameters, histograms and Gaussian distribution characteristics of the crop canopy reflectance data at different spatial scales before and after data correction with the proposed new method were shown in Table 2 and Figure 3. Two types of CDF curves in Figure 3 have the same meanings in Figure 2.

**Table 2:** Statistical characteristics of the crop canopy reflectance data at different spatial scales before and after data analysis and correction with the new method in Shunyi District and Changping District of Beijing, China.

Spectral range	Statistical parameters	IKONOS-Multispectral	HJ 1B-CCD2	HJ 1B-CCD2 (data correction)	MOD09A1	MOD09A1 (data correction)
Red						
	*μ*	0.065619	0.173539	0.070946	0.116622	0.065999
	*σ*	0.025418	0.025793	0.022803	0.009993	0.005612
	*σ* ^2^	0.000646	0.000665	0.000520	0.000100	0.000031
	CS	0.510546	−0.248537	0.069663	0.070507	−0.389323
	CK	−0.004755	−0.328195	0.615153	0.073495	−0.065596
Nir						
	*μ*	0.245903	0.314334	0.233396	0.233496	0.244387
	*σ*	0.066548	0.031453	0.055424	0.013501	0.020593
	*σ* ^2^	0.004429	0.000989	0.003072	0.000182	0.000424
	CS	0.179928	0.911457	−0.206560	−0.180333	1.348712
	CK	0.399334	2.368818	3.463020	−0.277366	1.987519

We concluded from Table 2 and Figure 3 that all of the red and near infrared reflectances of IKONOS-Multispectral, HJ 1B-CCD2, and MOD09A1 obeyed Gaussian distribution approximatively, though there existed some small differences among the statistical parameters of these Gaussian distributions. For CDF curves, there existed bigger differences among these three sequences of random variables, due to the differences of data amount. Then, the new proposed method was selected for these differences correction, with IKONOS-Multispectral data as small scale image, and HJ 1B-CCD2 and MOD09A1 as large scale images. According to the statistical parameters of the corrected reflectance datasets, the Gaussian distribution of satellite images at three different spatial scales were almost the same, and according to the CDF curves, the differences between the spatial distribution characteristics of multi-scale observations were reduced effectively. Taken near infrared reflectance of HJ 1B-CCD2 as an example, after data analysis and correction, its arithmetic mean value changed from 0.314334 to 0.233996, which was more closer to the arithmetic mean value of near infrared reflectance of IKONOS-Multispectral, i.e. 0.245903, and standard deviation value changed from 0.031453 to 0.055424, which was more closer to the standard deviation value of near infrared reflectance of IKONOS-Multispectral, i.e. 0.066548. And the CDF curve of corrected HJ 1B-CCD2 was more similar to the CDF curve of IKONOS-Multispectral. Also similar analysed results were achieved for red reflectance of HJ 1B-CCD2, and red and near infrared reflectances of MOD09A1. Above all, for this suburban underlying surface, which was also a non-homogeneous underlying surface, the new proposed method was also feasible and applicable for data correction, and after data processing with the new method, these multi-scale spatial reflectances had similar Gaussian distribution properties.

## Discussion

The current study demonstrates that the new proposed method integrated with parameter design into statistical model for data processing can quantitatively describe the differences between multi-source and multi-scale remote sensing observations, and effectively correct these differences based on probability theories and mathematical statistics. For the new proposed method, the key point for data correction is the standardization and transformation of Gaussian distribution, which can reveal and quantitatively describe the spatial relationship between small scale data (taken as baseline data) and large scale datasets. Experimental results validate the advantages of the proposed method in analysing and correcting differences between multi-scale datasets in underlying surfaces with different degrees of homogeneity, also confirm that the new data analysis and correction method not only enhances the correction precision between multi-scale datasets, but also reduces the differences both in homogeneous and non-homogeneous underlying surfaces. Above all, the new proposed method has a good prospect in the scientific research field for multi-scale observations analysing and correcting.

The pre-condition of using this new proposed method is Gaussian distribution properties of multiple remotely sensed observations, but actually the observations may deviate from Gaussian distributions because the processes of acquisition and pre-processing of multi-source and multi-scale spatial remote sensing data are influenced by soil and crop properties, atmospheric environments, performance of optical remote sensing systems, accuracy of radiometric calibration, atmospheric correction, and geometric calibration systems, degrees of homogeneity of the underlying surfaces, and data sizes. So, we select five statistical parameters and CDF for statistical characteristics extraction and Gaussian distribution test of these multi-scale spatial observed datasets. Then, if the datasets meet this pre-condition, the new proposed method can be conducted. Otherwise, before conducting multi-scale observations analysis and correction, we need to do data pre-process at first. Conducting a statistical test of the data firstly, and eliminating noise and outliers secondly, and doing data transformation finally, which can transform the original dataset into a Gaussian distributed one. Common data transformation methods include Logarithmic normal transformation, Rank-order transformation, and Multi-Gaussian transformation [18].

For this research, some limitations have to be noted. According to the new method, the main theoretical basis of the method are probability theories and mathematical statistics, so sufficient large of the data amount is necessary for data analysis and correction. We did not do research on how large of the data amount is enough in this study. In future, we can try to give a judgement method to decide whether the data amount is large enough or not for data correction, and how the differences of the data amount of these multiple observed datasets will affect data analysis and correction results from the aspects of geostatistics and landscape ecology. Another limitation of the study is that the data correction results depend on the baseline data, which means that the reliability, accuracy, and spatial scale of the baseline data have influences on the data correction results. The quantitative study of how the spatial scale of the baseline data affect the data correction results is necessary for analysing the feasibility and robustness of the new proposed data analysis and correction method. These problems in this research field need to be further studied, with the aim of qualitatively and quantitatively analysing the efficiency of new proposed method and reliability of its correction results. Also, in the following works, further researches of multi-scale crop growth monitoring and corresponding space consistency analysis should be done based on the original multiple datasets and corrected multiple datasets to quantitatively compare the differences of analysed results.

## Conclusion

In summary, the new proposed method can be applied for data analysis and correction of multi-source and multi-scale remote sensing observations both in homogeneous and non-homogeneous underlying surfaces. The new method involved the introduction of the statistical characteristics of multi-scale observations for data correction, which extended the availability of this method by considering the spatial and statistical relationships among multi-scale datasets. Also, the standardization and transformation of Gaussian distribution revealed the inherent links between observations at multiple spatial scales. In all, the new proposed method effectively extended the application field of the statistical models in data correction and enhanced the correction precision. Also, future work is needed to improve this method so that it can be effectively and pervasively applied in the research field of agricultural remote sensing for differences of multi-source and multi-scale spatial remote sensing observations analysing and correcting.

## References

[1] Liang SL (2007) Recent developments in estimating land surface biogeophysical variables from optical remote sensing. Progress in Physical Geography 31: 501–516.

[2] Wang JH, Zhao CJ, Huang WJ (2008) Basis and application of agriculture quantitative remote sensing. Science Press, 380 pp.

[3] Dong YY (2013) Analyzing spatial scale problems of crop growth parameters for growth monitoring with multi-scale remote sensing data. Zhejiang University: Phd Thesis, 210 pp.

[4] Qi J, Marsett RC, Moran MS, Goodrich DC, Heilman P, et al (2000) Spatial and temporal dynamics of vegetation in the San Pedro River basin area. Agricultural and Forest Meteorology 105: 55–68.

[5] He X, Jing YS, Gu XH, Huang WJ (2010) A province-scale maize yield estimation method based on TM and MODIS time-series interpolation. Sensor Letters 8: 2–5.

[6] Liu JD, Cao WB, Pei ZY, Guo L, Wu Q (2012) Fuzzy classification of arid and semi-arid region features using multi-resolution data. Transactions of the Chinese Society of Agricultural Engineering 28: 220–224.

[7] Wu H (2010) Study on scale effects and scaling method for land surface key parameters: case studies for leaf area index and surface temperature. Institute of Geographic Sciences and Natural Resources Research, CAS: Phd Thesis, 109 pp.

[8] BloČschl G (1999) Scaling issues in snow hydrology. Hydrological Processes 13: 2149–2175.

[9] Braswell BH, Hagen SC, Frolking SE, Salas WA (2003) A multivariable approach for mapping sub-pixel land cover distribution using MISR and MODIS: application in the Brazilian Amazon region. Remote Sensing of Environment 87: 243–256.

[10] Li X, Pan YC, Zhao CJ, Wang JH, Bao YS, et al (2005) Delineation and scale effect of precision agriculture management zones using yield monitor data over four years. Scientia Agricultura Sinica 38: 1825–1833.

[11] Tarnavsky E, Garrigues S, Brown ME (2008) Multiscale geostatistical analysis of AVHRR, SPOT-VGT, and MODIS global NDVI products. Remote Sensing of Environment 112: 535–549.

[12] Dong YY, Wang JH, Li CJ, Xu XG, Zhao JL, et al (2012) A new method for LAI spatial scaling based on Gaussian distribution theory. Advanced Materials Research 356: 2833–2837.

[13] Percival DP (1995) On estimation of the wavelet variance. Biometrika 82: 619–631.

[14] Wu JG, Jelinski DE, Luck M, Tueller PT (2000) Multiscale analysis of landscape heterogeneity: scale variance and pattern metrics. Geographic Information Sciences 6: 6–19.

[15] Liu ML, Tang XM, Liu JY, Zhuang DF (2001) Research on scaling effect based on 1km grid cell data. Journal of Remote Sensing 5: 183–190.

[16] Wackernagel H (2003) Multivariate geostatistics: an introduction with applications. Springer, 390 pp.

[17] Zhang TB, Tang JX, Liu DZ (2006) Feasibility of satellite remote sensing image about spatial resolution. Journal of Earth Sciences and Environment 28: 79–82.

[18] Webster R, Oliver MA (2007) Geostatistics for environmental scientists. John Wiley & Sons, 318 pp.

[19] Rosenqvist A, Shimada M, Watanabe M, Tadono T, Yamauchi K (2004) Implementation of systematic data observation strategies for ALOS PALSAR, PRISM and AVNIR-2. In: Geoscience and Remote Sensing Symposium, 2004. IGARSS’04. Proceedings. 2004 IEEE International. IEEE, volume 7, pp. 4527–4530.

[20] Vermote EF, Vermeulen A (1999) Atmospheric correction algorithm: spectral reflectances (MOD09). University of MaryLand.

[21] Tadono T, Shimada M, Murakami H, Takaku J (2009) Calibration of PRISM and AVNIR-2 onboard ALOS “Daichi”. IEEE Transactions on Geoscience and Remote Sensing 47: 4042–4050.

[22] Cooley T, Anderson GP, Felde GW, Hoke ML, Ratkowski AJ, et al.. (2002) FLAASH, a MODTRAN4-based atmospheric correction algorithm, its application and validation. In: Geoscience and Remote Sensing Symposium, 2002. IGARSS’02. 2002 IEEE International. IEEE, volume 3, pp. 1414–1418.

[23] Friedl MA, Brodley CE (1997) Decision tree classification of land cover from remotely sensed data. Remote Sensing of Environment 61: 399–409.

[24] Yang CC, Prasher SO, Enright P, Madramootoo C, Burgess M, et al (2003) Application of decision tree technology for image classification using remote sensing data. Agricultural Systems 76: 1101–1117.

[25] Rice JA (1995) Mathematical statistics and data analysis. Duxbury Press, 602 pp.

[26] Bárány I, Vu V (2007) Central limit theorems for Gaussian polytopes. The Annals of Probability 35: 1593–1621.

[27] Kallenberg O (2002) Foundations of modern probability. Springer, 664 pp.

